# Assessment of Practitioners’ Knowledge of the Diagnostic and Therapeutic Approach to Endo-Periodontal Lesions in Saudi Arabia

**DOI:** 10.7759/cureus.69619

**Published:** 2024-09-17

**Authors:** Hmoud Ali Algarni, Yasir Dilshad Siddiqui, Meshal Aber Alonazi, Muhammad Nadeem Baig, Ammar Ahmed Siddiqui, Mohammed Sulaiman Alaenazi, Azhar Iqbal, Farooq Ahmad Chaudhary

**Affiliations:** 1 Department of Restorative Dental Sciences, College of Dentistry, Jouf University, Sakaka, SAU; 2 Department of Preventive Dental Sciences, College of Dentistry, Jouf University, Sakaka, SAU; 3 Department of Preventive Dental Sciences, College of Dentistry, University of Hail, Hail, SAU; 4 Ministry of Health, Specialized Dental Center, Arar, SAU; 5 School of Dentistry, Shaheed Zulfiqar Ali Bhutto Medical University, Islamabad, PAK

**Keywords:** diagnosis, endo-periodontal lesions, knowledge, practitioners, treatment

## Abstract

Introduction: Endodontic-periodontal diseases frequently pose significant challenges for clinicians in terms of diagnosis, management, and prognosis. Practitioners' knowledge of endo-periodontal lesion (EPL) diagnosis is essential for formulating appropriate treatment plans. This study aimed to evaluate the knowledge of diagnostic and therapeutic approaches to EPLs among dental practitioners in Saudi Arabia.

Methods: The study used a cross-sectional survey among Saudi Arabian dental practitioners using a self-administered questionnaire. The statistical methods used included descriptive analysis, Pearson's chi-square test, Fisher's exact test, and binary logistic regression.

Results: The study included 312 respondents, with 95 (30.4%) females and 217 (69.6%) males. The majority were aged 25 to 30 (n = 155, 49.7%). Significant associations were found between participant knowledge and the following variables: age (P < 0.001), organization (P = 0.005), employment (P = 0.001), professional experience (P < 0.001), postgraduate degree (P = 0.002), specialty (P < 0.001), Saudi Commission for Health Specialities (SCHS) rank (P = 0.004), and continuing training (P = 0.043). Additionally, individuals aged 36-40 were 4.3 times more likely to have good knowledge compared to those aged 25-30 (adjusted odds ratio [AOR] = 4.27, P = 0.020), and lecturers were 4.4 times more likely to have good knowledge compared to interns (AOR = 4.40, P = 0.054).

Conclusion: This indicates that the practitioners' overall knowledge was above average and that they possess a fundamental understanding of EPLs. These findings have valuable effects on clinical practice, suggesting that evidence-based guidelines and targeted educational programs can enhance the practitioners’ diagnostic accuracy and treatment strategies for EPLs, ultimately improving patient outcomes and clinical efficacy.

## Introduction

The periodontium and dental pulp share morphological, functional, and developmental connections, establishing a close relationship [1,2]. The impact of periodontal disorders on pulp tissue was first documented a century ago [3]. In 1964, "retrograde periodontitis" was identified wherein pulpal illness induces a condition affecting the periodontium [4]. They described this as a scenario where pathogens originating from the pulp affect the periodontium, potentially exacerbating or hindering the healing of periodontal diseases. These authors noted that these two processes often exhibit similar signs and symptoms, complicating their differentiation [4].

Traditionally, endodontic and periodontal lesions are classified based on the origin of the infection: primary endodontic lesions, primary periodontal lesions, and various combinations of these [2,5,6]. Despite the appearance that the periodontium and the dental pulp are two separate tissues, there are a variety of possible pathways for communication between these two tissues [7]. These pathways include the apical foramen [2], exposed dentinal tubules [8,9], lateral and accessory canals [10], specific anatomical variations [11], and pathological conditions such as root fractures and perforations [12].

A thorough diagnostic process can differentiate between endodontic, periodontal, or mixed-origin lesions [1,13,14]. The classification of endo-periodontal lesions (EPLs) is extensively debated in the literature, posing challenges in diagnosis, treatment, and prognosis for clinicians [1,15]. While symptoms of endodontic and periodontal diseases are typically distinguishable, clinical and radiographic findings can occasionally lead to confusion, complicating the final diagnosis and potentially resulting in suboptimal treatment decisions, sometimes necessitating tooth extraction [16,17]. The complexity of EPLs impacts all branches of dentistry, emphasizing the necessity of a multidisciplinary approach to enhance the likelihood of successful outcomes in diagnosis and treatment [18].

Dental care has evolved rapidly in recent years in Saudi Arabia. However, it remains unclear how well dental professionals are equipped to diagnose and manage EPLs effectively. Understanding the knowledge, practices, and potential gaps in the clinical management of EPLs among Saudi dental practitioners is essential for improving patient outcomes and developing targeted training programs and clinical guidelines. Therefore, the present study aims to assess the practitioners’ knowledge of the diagnostic and therapeutic approaches to EPLs in Saudi Arabia. This will allow healthcare authorities to understand and ensure that dental professionals are fully equipped to deliver the highest standard of care, ultimately benefiting both patients and the healthcare system as a whole.

## Materials and methods

The study received approval from the Research Ethics Committee of the University of Hail, Saudi Arabia (Reference No. H-2024-024). It was an observational study with a descriptive cross-sectional design conducted among registered dental professionals in Saudi Arabia, regardless of their gender or area of practice. Data collection took place from April 2024 to June 2024. The sample size was calculated using the total number of registered dental professionals in Saudi Arabia, which is 42,906. The calculation assumed a response distribution of 50%, a confidence level of 90%, and a margin of error of 5%. Using these parameters, the required sample size for this study was determined to be 279 participants. To account for potential refusals and incomplete responses, the plan was to recruit 310 subjects. This sample size ensures adequate power to assess the knowledge of practitioners regarding the diagnostic and therapeutic approaches to EPLs. Due to logistical constraints, a non-probability snowball sampling technique was employed to gather data using an online Google form. The questionnaire was disseminated through various digital platforms, including WhatsApp, Facebook, and email. Participants were encouraged to forward the questionnaire to other dental professionals, making it unfeasible to track the exact number of individuals who received the survey. However, by the end of the data collection phase, responses from 312 dental professionals had been obtained. All participants were informed about the study's purpose and assured that their participation was voluntary and written informed consent was taken. Confidentiality and anonymity were strictly maintained, with no personally identifiable information included in the data processing or analysis.

The current study utilized a self-administered questionnaire adapted from a previous study by Kabore et al. [1]. To confirm the questionnaire was related to the local context, it was reviewed by a panel of experts familiar with the dental healthcare system in Saudi Arabia. Some modifications were made to reflect region-specific terminologies and practices. Additionally, some questions were reworded to simplify complex terms, ensuring clarity for professionals with varying levels of expertise. The questionnaire was then pilot-tested with a small group of local dental professionals to confirm its clarity and applicability. Based on the feedback from the pilot test, minor adjustments were made to improve the comprehensibility of the questions. To validate the questionnaire, five endodontics and periodontic experts assessed the questionnaire for relevance and appropriateness in evaluating EPL knowledge. Internal consistency was confirmed with a Cronbach’s alpha of 0.85, indicating good reliability.

The questionnaire was divided into three sections: (1) Participant Sociodemographic: This section collected data on age, gender, years of professional experience, formal postgraduate training, type of continuing education, and practice sector. (2) Diagnostic Knowledge of EPLs: This section included six questions focusing on knowledge of endodontic-periodontal communication pathways, clinical and functional signs, criteria for evaluating mixed or true EPLs, relevant pathologies, types of radiographs used, and characteristic radiographic signs. (3) Prognostic and Therapeutic Knowledge of EPLs: This section also contained six questions, addressing criteria for deciding whether to preserve a tooth affected by EPLs, methods for managing different clinical presentations, success rate standards, and postoperative follow-up schedules.

Additionally, two scenario-based questions were included to assess the practitioner's diagnostic and therapeutic interpretation of a clinical case involving the radiograph and clinical image of a tooth.

To gauge the level of awareness regarding EPLs, scores were generated based on responses to the questionnaire. Each "Yes" response was assigned a score of 1, and each "No" response was assigned a score of 0. The expected values for the partial diagnostic knowledge score, partial therapeutic score, and overall knowledge score were 17, 15, and 32, respectively. Based on these scores, respondents were categorized into three knowledge levels: Good Knowledge (score of more than 70%), Average Knowledge (score between 50% and 70%), and Poor Knowledge (score of less than 50%).

The initial data analysis focused on cleaning the dataset to address missing values and incorrect entries. Statistical methods used included descriptive analysis, and Pearson's chi-square test was used to assess associations between participants' sociodemographic characteristics and their knowledge of diagnostic, therapeutic, and prognostic approaches to EPLs. Fisher’s exact test was used when more than 20% of the expected counts were below 5, and logistic regression was applied when deemed appropriate. Simple logistic regression was performed to obtain crude odds ratios (COR) for predictors of good knowledge in partial diagnostic and therapeutic areas. Predictors with P-values less than 0.25 were included in a multiple logistic regression to determine adjusted odds ratios (AOR). The multiple logistic regression employed both forward and backward likelihood ratio (LR) methods, with the final model being established using the enter method. All statistical analyses were conducted using SPSS version 27 (IBM Corp., Armonk, NY).

## Results

Table 1 displays the sociodemographic characteristics of the study participants. The study included 312 respondents, with 95 (30.4%) females and 217 (69.6%) males. The majority were aged 25 to 30 (49.7%), worked in government (88.5%), had 0-5 years of experience (58.7%), held postgraduate degrees (55.5%), were general practitioners (24.3%), specialists in endodontics (19.9%), had other types of Saudi Commission for Health Specialities (SCHS) rank (33.6%), and participated in continuous training (80.8%).

**Table 1:** Sociodemographic characteristics (n = 312) The data has been represented as N (%). N = number.

Variable	Category	n (%)
Gender		
	Female	95 (30.4)
	Male	217 (69.6)
Age		
	25-30	155 (49.7)
	31-35	64 (20.5)
	36-40	39 (12.5)
	41-45	27 (8.7)
	46-50	15 (4.8)
	>05	12 (3.8)
Organization		
	Government	276 (88.5)
	Private	5 (1.6)
	Semi-government	15 (4.8)
	Others	16 (5.1)
Employment		
	Intern	89 (28.5)
	Lecturer	46 (14.7)
	Assistant Professor	56 (17.9)
	Professor	4 (1.3)
	NA	117 (37.5)
Professional experience		
	0-5	183 (58.7)
	6-10	54 (17.3)
	11-15	19 (6.1)
	16-20	32 (10.3)
	>20	24 (7.7)
Postgraduate (n = 301)		
	No	134 (44.5)
	Yes	167 (55.5)
Specialty		
	Dental public health	3 (1.0)
	Endodontics	62 (19.9)
	Forensic dentistry	3 (1.0)
	General practitioner	76 (24.4)
	Maxillofacial	24 (7.7)
	Operative dentist	15 (4.8)
	Oral medicine	11 (3.5)
	Oral pathologist	9 (2.9)
	Orthodontist	9 (2.9)
	Periodontics	23 (7.4)
	Prosthodontist	20 (6.4)
	None	57 (18.3)
Rank in SCHS		
	Consultant	34 (10.9)
	General dental practitioner	68 (21.8)
	Registrar	73 (23.4)
	Senior registrar	32 (10.3)
	Others	105 (33.7)
Continuing training		
	No	60 (19.2)
	Yes	252 (80.8)

Table 2 details the practitioners' knowledge of the diagnostic approach to EPLs based on their responses to each question. The results indicate that practitioners achieved the highest average percentage for the question, "What types of radiography do you carry out?" with an average score of 76.17%, and the majority correctly selected the periapical option (95.5%). Conversely, the lowest average percentage was for the question "What type of periodontal probing allows a true EPL to be characterized?" with an average score of 49.85%. Notably, more than half of the practitioners correctly chose "yes" for circumferential probing as a "U" (57.4%). Table 3 provides information about the practitioner's understanding of the clinical case involving the radiographic findings of a deep carious lesion.

**Table 2:** Practitioner's knowledge of the diagnostic approach to EPLs based on each response The data has been represented as N (%). EPLs = endo-periodontal lesions; N = number.

Parameter	Option	No (%)	Yes (%)	Overall %
Does an endodontic or periodontal lesion induce an EPL by means of?				68.27
	Apical foramen	14 (4.5)	298 (95.5)	
	Lateral root canal	39 (12.5)	273 (87.5)	
	Root perforation	44 (14.1)	268 (85.9)	
	Periodontal pocket	59 (18.9)	253 (81.1)	
	Dentinal tubule	181 (58.0)	131 (42.0)	
	Endodontic access cavity	147 (47.1)	165 (52.9)	
	Root resorption	121 (38.8)	191 (61.2)	
What are the signs to consider regarding the occurrence of EPLs?				59.56
	Pulp necrosis	48 (15.4)	264 (84.6)	
	Swelling	33 (10.6)	279 (89.4)	
	Abscess	35 (11.2)	277 (88.8)	
	Pain	51 (16.3)	261 (83.7)	
	Tooth mobility with deep periodontal pocket	36 (11.5)	276 (88.5)	
	Negative pulp vitality test	80 (25.6)	232 (74.4)	
	Food impaction	150 (48.1)	162 (51.9)	
	Coronal fracture	181 (58.0)	131 (42.0)	
What type of periodontal probing allows a true EPL to be characterized?				49.85
	Circumferential as a "V”	132 (42.3)	180 (57.7)	
	Circumferential as a "U"	133 (42.6)	179 (57.4)	
What pathologies allow a differential diagnosis of EPLs?				70.30
	Root cracks and fractures	30 (9.6)	282 (90.4)	
	Coronal cracks or fractures	170 (54.5)	142 (45.5)	
	Retropulpitis	70 (22.4)	242 (77.6)	
	Dental anatomical anomalies	129 (41.3)	183 (58.7)	
What types of radiography do you carry out?				76.17
	Periapical	14 (4.5)	298 (95.5)	
	Bitewing	129 (41.3)	183 (58.7)	
	Periapical with a GP point in the periodontal pocket or the fistula	26 (8.3)	286 (91.7)	

**Table 3:** Case scenario The data has been represented as N (%). N = number.

Radiograph showing the presence of deep coronal restoration and mesial caries approaching the pulp with periapical radiolucency, confirming	
Responses	Overall N (%)
Combined lesion	80 (25.6)
Primary endodontic lesion	64 (20.5)
Primary endodontic secondary periodontic lesion	136 (43.6)
Primary periodontic lesion	32 (10.3)

Table 4 outlines the practitioners' knowledge of the therapeutic and prognostic approaches to EPLs based on their responses to each question. The results indicate that practitioners scored the highest average percentage for the question "Is the level of healing obtained due to the following?" with an average score of 88.65%, and the majority correctly selected "control of the risk factors" (96.2%). The lowest average percentage was for the question "Does healing of an EPL of endodontic origin require the following?" with an average score of 68.37%, although most practitioners correctly chose "endodontic treatment and periodontal monitoring" (92.3%). Table 5 presents the practitioner's therapeutic interpretation of the clinical case involving the radiograph and clinical image of a tooth with a grade III furcation involvement, an 8 mm periodontal pocket, and deep dental caries.

**Table 4:** Practitioner's knowledge of the therapeutic and prognostic approaches to EPLs based on each response The data has been represented as N (%). EPLs = endo-periodontal lesions; N = number.

Parameter	Option	No (%)	Yes (%)	Overall %
Is the decision to conserve or to extract a tooth afflicted by EPL linked to the following?				72.75
	Degree of affliction of the tooth	40 (12.8)	272 (87.2)	
	Motivation of the patient	62 (19.9)	250 (80.1)	
	Situation of the tooth	28 (9.0)	284 (91.0)	
	Reason for consultation	102 (32.7)	210 (67.3)	
Does healing of an EPL of endodontic origin require the following?				68.37
	Endodontic treatment only	218 (69.9)	94 (30.1)	
	Endodontic treatment and periodontal monitoring	24 (7.7)	288 (92.3)	
	Periodontal treatment only	258 (82.7)	54 (17.3)	
Does healing of an EPL of periodontal origin require the following?				76.37
	Endodontic treatment only	254 (81.4)	58 (18.6)	
	Endodontic and periodontic treatment	42 (13.5)	270 (86.5)	
	Periodontal treatment only associated with assessment of the pulp vitality	121 (38.8)	191 (61.2)	
Does healing of an EPL of mixed origin require the following?				82.07
	Endodontic treatment only	235 (76.1)	74 (23.9)	
	Endodontic and periodontic treatment	27 (8.7)	285 (91.3)	
	Periodontal treatment only	246 (78.8)	66 (21.2)	
Is the level of healing obtained due to the following?				88.65
	Quality of the treatment	21 (6.7)	291 (93.3)	
	Degree of motivation of the patient	84 (26.9)	228 (73.1)	
	Control of the risk factors	12 (3.8)	300 (96.2)	
	Accuracy of the diagnosis	25 (8.0)	287 (92.0)	
When do you clinically and radiographically evaluate the healing of EPLs?				77.77
	Immediately	235 (75.3)	77 (24.7)	
	3 months	64 (20.5)	248 (79.5)	
	6 months	67 (21.5)	245 (78.5)	

**Table 5:** Case scenario The data has been represented as N (%). N = number.

A 32-year-old woman reported experiencing pain in the lower right back region of her tooth for the past eight days. Her medical and dental history did not provide any relevant information. Upon intraoral examination, it was observed that tooth #37 had deep dental caries and was sensitive to both horizontal and vertical percussion. The tooth also showed signs of pulp necrosis upon testing, and tooth #36 was found to be missing. Further examination revealed a deep periodontal pocket of 8 mm on the buccal side and 10 mm on the lingual side in relation to tooth #37. Using a Nabers probe, a grade III furcation defect was identified according to Glickman's classification of furcation involvement. Patient insists to save her tooth. What would be the appropriate treatment for this patient?	
Response	Overall N (%)
Endodontic therapy followed by surgical periodontal therapy	261 (83.7)
Endodontic therapy only	3 (1.0)
Extraction	25 (8.0)
Periodontal therapy followed by endodontic therapy	23 (7.4)

Table 6 presents practitioners' knowledge of the diagnostic approach to EPLs. The results indicate that the mean scores for all five questions related to practitioners' knowledge of the diagnostic approach to EPLs were above the expected middle value.

**Table 6:** Practitioner's knowledge of the diagnostic approach to EPLs The data has been represented as mean (SD). EPLs = endo-periodontal lesions; N = number; SD = standard deviation.

Question	N	Mean (SD)	Range in data	Possible range
What are the causes of development of an endodontic or periodontal lesion?	312	4.78 (1.02)	1-7	0-7
What are the signs to consider regarding the occurrence of EPLs?	312	5.48 (1.19)	2-8	0-8
What type of periodontal probing allows a true EPL to be characterized?	312	1.00 (0.84)	0-2	0-2
What pathologies allow a differential diagnosis of EPLs?	312	2.81 (0.84)	1-4	0-4
What types of radiography do you carry out?	312	2.29 (0.64)	0-3	0-3

Table 7 presents practitioners' knowledge of the therapeutic and prognostic approaches to EPLs. The results indicate that the mean scores for all questions exceeded the expected middle value.

**Table 7:** Practitioner's knowledge of the therapeutic and prognostic approaches to endo-periodontal lesions The data has been represented as mean (SD). EPLs = endo-periodontal lesions; N = number; SD = standard deviation.

Question	N	Mean (SD)	Range in data	Possible range
Is the decision to conserve or to extract a tooth afflicted by EPL linked to the following?	312	2.91 (0.78)	0-4	0-4
Does healing of an EPL of endodontic origin require the following?	312	2.05 (0.46)	0-3	0-3
Does healing of an EPL of periodontal origin require the following?	312	2.29 (0.60)	1-3	0-3
Does healing of an EPL of mixed origin require the following?	309	2.46 (0.88)	0-3	0-3
Is the level of healing obtained due to the following?	312	3.54 (0.75)	1-4	0-4
When do you clinically and radiographically evaluate the healing of EPLs?	312	2.33 (0.71)	1-3	0-3

Table 8 shows the association between sociodemographic characteristics and participant knowledge. The results reveal significant associations between participant knowledge and the following variables: age (P < 0.001), organization (P = 0.005), employment (P = 0.001), professional experience (P < 0.001), postgraduate degree (P = 0.002), specialty (P < 0.001), SCHS rank (P = 0.004), and continuing training (P = 0.043).

**Table 8:** The association between sociodemographic characteristics and participants’ knowledge The data has been represented as N (%). P <0.05 is considered statistically significant. N = number; DF = degree of freedom.

Variable		Poor n (%)	Average n (%)	Good n (%)	Chi-square (DF)	P-value
Gender					3.29 (2)	0.182
	Female	0 (0)	12 (22.8)	71 (77.2)		
	Male	7 (3.2)	56 (25.8)	154 (71.0)		
Age					47.15 (10)	<0.001
	25-30	7 (4.5)	60 (38.7)	88 (56.8)		
	31-35	0 (0)	12 (18.8)	52 (81.3)		
	36-40	0 (0)	5 (12.8)	34 (87.2)		
	41-45	0 (0)	0 (0)	24 (100)		
	46-50	0 (0)	0 (0)	15 (100)		
	>05	0 (0)	0 (0)	12 (100)		
Organization					31.45 (6)	0.005
	Government	4 (1.5)	63 (23.1)	206 (75.5)		
	Semi-government	0 (0)	0 (0)	5 (100)		
	Private	0 (0)	7 (46.7)	8 (53.3)		
	Others	3 (18.8)	7 (43.8)	6 (37.5)		
Employment					48.59 (8)	0.001
	Intern	7 (7.9)	33 (37.1)	49 (55.1)		
	Lecturer	0 (0)	3 (7.0)	40 (93.0)		
	Assistant Professor	0 (0)	10 (17.9)	46 (82.1)		
	Professor	0 (0)	4 (100)	0 (0)		
	NA	0 (0)	27 (23.1)	90 (76.9)		
Professional experience					40.97 (8)	<0.001
	0-5	7 (3.8)	64 (35.0)	112 (61.2)		
	6-10	0 (0)	13 (24.1)	41 (75.9)		
	11-15	0 (0)	0 (0)	16 (100)		
	16-20	0 (0)	0 (0)	32 (100)		
	>20	0 (0)	0 (0)	24 (100)		
Postgraduate (n = 301)					11.80 (2)	0.002
	No	3 (2.2)	46 (34.3)	85 (63.4)		
	Yes	4 (2.4)	28 (17.1)	132 (80.5)		
Specialty					139.21 (22)	<0.001
	Dental public health	0 (0)	0 (0)	3 (100)		
	Endodontics	0 (0)	4 (6.5)	58 (93.5)		
	Forensic dentistry	0 (0)	3 (100)	0 (0)		
	General practitioner	0 (0)	34 (44.7)	42 (55.3)		
	Maxillofacial	7 (29.2)	6 (25.0)	11 (45.8)		
	Operative dentist	0 (0)	3 (20.0)	12 (80.0)		
	Oral medicine	0 (0)	0 (0)	8 (100)		
	Oral pathologist	0 (0)	0 (0)	9 (100)		
	Orthodontist	0 (0)	4 (44.4)	5 (55.6)		
	Periodontics	0 (0)	5 (21.7)	18 (78.3)		
	Prosthodontist	0 (0)	0 (0)	20 (100)		
	None	0 (0)	18 (31.6)	39 (68.4)		
Rank in SCHS					22.69 (8)	0.004
	Consultant	0 (0)	0 (0)	34 (100)		
	General dental practitioner	0 (0)	23 (33.8)	45 (66.2)		
	Registrar	4 (5.5)	22 (30.1)	47 (64.4)		
	Senior registrar	0 (0)	8 (25.0)	24 (75.0)		
	Others	3 (2.9)	24 (23.5)	75 (73.5)		
Continuing training					6.57 (2)	0.043
	No	0 (0)	8 (14.0)	49 (86.0)		
	Yes	7 (2.8)	69 (27.4)	176 (69.8)		

For age, individuals aged 41 years and older (100%) have a higher proportion of good knowledge. Regarding organizations, those working in government (75.5%) and semi-government (n = 5, 100%) demonstrate a higher proportion of good knowledge. For employment, the lecturers (93.0%) and associate professors (82.1%) demonstrate a higher proportion of good knowledge. For professional experience, those with 11 years and above demonstrate a higher proportion of good knowledge (100%). Those with a postgraduate degree demonstrate a higher proportion of good knowledge (80.5%). For specialties, those in oral medicine (100%), oral pathology (100%), and prosthodontists (100%) demonstrate a higher proportion of good knowledge. Those with a postgraduate degree demonstrate a higher proportion of good knowledge (80.5%).

Among the different specialties, a total of 62 participants (19.9%) were endodontists, representing the highest number of participants in any specialty and demonstrating a high proportion of good knowledge (93.5%). Other specialties, such as operative dentistry, periodontology, prosthodontics, and oral medicine, also showed high levels of good knowledge: 80% among 15 operative dentists, 78.3% among 23 periodontists, 100% among 20 prosthodontists, and 100% among eight oral medicine specialists.

The logistic regression analysis presented in Table 9 shows that the final model identified two factors - age group and employment - as significant predictors of good knowledge. For age group, the 31-35-year-olds were 72% more likely to have good knowledge compared to the 25-30-year-olds (AOR = 1.72, P = 0.233), and the 36-40-year-olds were 4.3 times more likely to have good knowledge compared to the 25-30-year-olds (AOR = 4.27, P = 0.020). For employment, the lecturers were 4.4 times more likely to have good knowledge compared to the interns (AOR = 4.40, P = 0.054).

**Table 9:** Factors associated with the participants' knowledge using simple and multiple logistic regression analyses The data has been represented as odds ratio (95% CI). P <0.05 is considered statistically significant. COR = crude odds ratio; AOR = adjusted odds ratio; CI = confidence interval; DF = degree of freedom.

Variable		COR	P-value	AOR	Wald (DF)	P-value
Gender						
	Female	1				
	Male	0.72 (0.41, 1.28)	0.263			
Age						
	25-30	1		1		
	31-35	3.30 (1.63, 6.67)	0.001	1.72 (0.70, 4.21)	1.42 (1)	0.233
	36-40	5.18 (1.92, 13.95)	0.001	4.27 (1.26, 14.47)	5.41 (1)	0.020
	41-45	-	-	-		-
	46-50	-	-	-		-
	>05	-	-	-		-
Organization						
	Government	1				
	Semi-government	-	-	-		-
	Private	0.37 (0.13, 1.06)	0.065	-		-
	Others	0.20 (0.07, 0.56)	0.002	-		-
Employment						
	Intern	1		1		
	Lecturer	10.88 (3.13, 37.82)	<0.001	4.40 (0.97, 19.94)	3.70 (1)	0.054
	Assistant Professor	3.76 (1.69, 8.37)	0.001	0.92 (0.32, 2.68)	0.02 (1)	0.882
	Professor	-	-	-	0.00	-
	NA	2.72 (1.49, 4.96)	0.001	1.50 (0.76, 2.99)	1.35 (1)	0.246
Professional experience						
	0-5	1				
	6-10	2.00 (1.01, 3.99)	0.049	-		-
	11-15	-	-	-		-
	16-20	-	-	-		-
	>20	-	-	-		-
Postgraduate (n = 301)						
	No	1				
	Yes	2.38 (1.41, 4.01)	0.001	-		-
Continuing training						
	No	1				
	Yes	0.38 (0.17, 0.84)	0.016	-		-

## Discussion

This study was a cross-sectional survey of dental practitioners in Saudi Arabia, utilizing self-administered questionnaires to evaluate their knowledge of the diagnostic and therapeutic approaches to EPLs. The results of this study highlight a strong foundation of knowledge among the participants, in terms of their diagnostic and therapeutic approaches to EPLs. The results indicate that practitioners scored highest on the question "What types of radiography do you perform?" with an average score of 76.17%, and most correctly identified the periapical option (95.5%). According to a recent study by Kabore et al. (2022) [1], 62% of dental surgeons prescribed a diagnostic periapical X-ray with a gutta-percha point placed within the periodontal pocket or ostium surrounding the fistula. As a result, the professionals who participated in the interviews possess a very strong understanding of the supplementary radiographic examination. Furthermore, this method can assist professionals in determining the origin of inflammation and discriminating between the periodontal and endodontic genesis of diseases [19]. This high level of knowledge is crucial for ensuring precise diagnostics and guiding effective treatment plans for EPLs.

Concerning practitioners' knowledge of the therapeutic and prognostic approaches to EPL, the results show the highest understanding regarding the level of healing with an average score of 88.65%. The majority correctly identified "control of the risk factors" as a means to promote the healing process (96.2%). Recent research has demonstrated that an important consideration in the choice of treatment is the patient's motivation [20,21]. This drive stems from the patient's viewpoint regarding the anticipated cosmetic results of the procedure, as well as the prices, time frame, and durability of the many treatment alternatives. In a study by Kabore et al. (2022) [1], 24% of the practitioners acknowledged that they would handle endodontic therapy by themselves. In fact, in the gingival-dental groove, a purulent discharge externalizes from a periodontal pocket with five bony walls when periodontal diseases are in an acute phase [1,22,23]. Following endodontic treatment, they may spontaneously recover [2,24-26].

The results indicate that the mean scores for all questions related to practitioners’ knowledge of the diagnostic and therapeutic approaches to EPLs were above their expected midpoint. This demonstrates that the practitioners' overall knowledge was above average and that they possess a basic understanding of EPLs. This is consistent with a previous study, which found that 70% of the practitioners had above-average knowledge of diagnosis and 98% had above-average knowledge of the therapeutic and prognostic approaches to EPL [1]. These results highlight that the dental practitioners surveyed possess an overall good level of knowledge, which is key to ensuring accurate diagnosis and successful therapeutic outcomes. The accuracy of the diagnosis and an informed choice of the appropriate course of treatment are key factors in therapeutic success [13]. Thus, the key to the effectiveness of EPL interventions is a comprehensive understanding of the diagnostic and therapeutic techniques.

The results reveal significant associations between participants' overall knowledge of EPL and sociodemographic factors, including age, organization, employment, professional experience, postgraduate degree, specialty, SCHS rank, and continuing training. Specifically, individuals aged 41 years and older (100%), lecturers (93.0%), and those with 11 years or more of professional experience (100%). This reflects that experience and continuous professional development greatly contribute to expertise in managing EPL cases. Further among the different specialties, a total of 62 participants (19.9%) were endodontists, representing the highest number of participants in any specialty and demonstrating a high proportion of good knowledge (93.5%). Other specialties, such as operative dentistry, periodontology, prosthodontics, and oral medicine, also showed high levels of good knowledge: 80% among 15 operative dentists, 78.3% among 23 periodontists, 100% among 20 prosthodontists, and 100% among eight oral medicine specialists. However, due to the smaller number of participants in these other specialties, the percentage of correct answers may not be fully representative. Only a few studies have evaluated the relationship between practitioner's knowledge of diagnostic and therapeutic EPL and demographic characteristics. These disparities demonstrate that, in order to guarantee the best probability of providing effective therapy, an appropriate EPL therapy requires an in-depth understanding of numerous areas of dentistry [27]. Such interdisciplinary knowledge is crucial, as the management of EPLs often requires comprehensive understanding across various domains of dentistry.

Furthermore, the study findings indicate that age group and employment are significant predictors of adequate knowledge, with older individuals and those in the teaching profession being more likely to possess adequate knowledge of diagnostic and therapeutic EPL. Due to their greater years of clinical expertise, older practitioners are better equipped to handle a wider range of situations, including EPLs. This practical experience greatly enhances their knowledge and expertise [28]. Teachers often undertake research, participate in ongoing training, and keep up with the most recent developments in their teaching sector. Their dedication to lifelong learning enables individuals to preserve and improve their body of knowledge [29,30].

In this study, Pearson’s chi-square test was used to examine associations between participants’ sociodemographic characteristics and their knowledge of diagnostic and therapeutic approaches to EPLs, as it is suitable for categorical data. Logistic regression was chosen to identify predictors of good knowledge, given the binary nature of the outcome variables (e.g., good vs. average/poor knowledge). Simple logistic regression provided COR, while multiple logistic regression was used to adjust for confounding factors and determine AORs. These tests helped uncover key demographic influences on practitioner knowledge, guiding potential interventions and training.

There are limitations to this study that should be considered. First, the use of self-administered questionnaires can introduce response bias, as participants may overestimate their knowledge or provide socially desirable answers. Furthermore, without the ability to clarify questions in real time, there is a possibility of misinterpretation, which can affect the accuracy of the responses. Second, the non-probability snowball sampling technique used in this study may introduce selection bias, as participants were encouraged to share the survey with peers, potentially leading to a sample that is not fully representative of the broader population of dental practitioners in Saudi Arabia. These biases limit the generalizability of the findings and should be considered when interpreting the results.

Despite these limitations, the findings of this study have significant implications for clinical practice and future research. The study highlights key areas of strength in the diagnostic and therapeutic knowledge of EPLs among dental practitioners, while also identifying potential gaps that could be addressed through targeted training. The insights gained from this research can be used to inform the development of evidence-based guidelines for the diagnosis and management of EPLs, particularly in Saudi Arabia. Moreover, the results suggest that there is a need for continuous professional development programs tailored to different specialties and levels of experience. Implementing structured training programs can ensure that practitioners remain up to date with the latest diagnostic tools and treatment protocols, ultimately improving patient care. Future research can build upon these findings by exploring the effectiveness of such programs and assessing how standardized guidelines can be adopted in clinical practice to enhance treatment outcomes.

## Conclusions

Endodontic-periodontal diseases often pose significant challenges to clinicians in terms of diagnosis, management, and prognosis. Therefore, practitioners' knowledge of EPL diagnosis is crucial to ensuring appropriate treatment plans. The study reveals that practitioners' overall knowledge was above average and that they possess a fundamental understanding of EPLs. Additionally, the findings indicate that age group and employment are significant predictors of adequate knowledge, with older individuals and those in the teaching profession being more likely to have sufficient knowledge of diagnostic and therapeutic approaches to EPL.

To address knowledge gaps, we recommend developing targeted continuing education programs, implementing standardized clinical guidelines, and encouraging mentorship programs between experienced and younger practitioners. These measures can enhance diagnostic accuracy, treatment consistency, and overall patient outcomes in EPL management.
